# Ki67 expression and the effect of neo-adjuvant chemotherapy on luminal HER2-negative breast cancer

**DOI:** 10.1186/1471-2407-14-550

**Published:** 2014-07-30

**Authors:** Yoshiya Horimoto, Atsushi Arakawa, Masahiko Tanabe, Hiroshi Sonoue, Fumie Igari, Koji Senuma, Emi Tokuda, Hideo Shimizu, Taijiro Kosaka, Mitsue Saito

**Affiliations:** Department of Breast Oncology, Juntendo University School of Medicine, 2-1-1 Hongo, Bunkyo-ku, 113-0033 Tokyo, Japan; Department of Human Pathology, Juntendo University School of Medicine, 2-1-1 Hongo, Bunkyo-ku, 113-0033 Tokyo, Japan

**Keywords:** Ki67, Cut-off value, Luminal breast cancer, Neo-adjuvant chemotherapy, Pathological complete response

## Abstract

**Background:**

Patients with luminal HER2-negative tumours have a favourable prognosis. However, there is a subpopulation in which poorer outcomes are obtained with endocrine therapy alone. This subpopulation is considered to benefit from chemotherapy. However, the significance of chemotherapy for those with luminal tumours has decreased due to recent changes in treatment strategies. Thus, it is often difficult to determine whether we should recommend chemotherapy to such patients in clinical practice. We investigated Ki67 expression, as a means of predicting the responses of luminal HER2-negative breast cancer patients to neo-adjuvant chemotherapy (NAC), in order to identify a subpopulation that would benefit from these treatments.

**Methods:**

We enrolled 114 luminal HER2-negative breast cancer patients undergoing surgery after NAC. Biomarkers were examined using biopsy specimens obtained prior to treatment, to avoid any chemotherapy-related effects. Chemotherapy effects were determined employing operative specimens and we defined pathological complete response (pCR) as invasive nest disappearance, based only on the primary breast tumour. We applied receiver operating characteristic curve analysis to data from our 114 patients, to investigate Ki67 expression as a predictor of pCR.

**Results:**

The pCR rate was significantly higher for tumours with high Ki67 expression (p < 0.01) and all patients who obtained pCR remained recurrence-free during the median 58-month observation period. We identified 35% as the Ki67 cut-off value which distinguishes those with a pCR from other cases. Another dataset, comprised of 196 patients with a median 29-month observation period, was recruited for validation. Disease-free survival was found to be significantly (p < 0.01) lower in the patients with tumours in which Ki67 expression was higher than 35%.

**Conclusion:**

Our results raise the possibility of the luminal HER2-negative subpopulation with Ki67 expression higher than 35% benefiting from chemotherapy, as evidenced by improved survival.

**Electronic supplementary material:**

The online version of this article (doi:10.1186/1471-2407-14-550) contains supplementary material, which is available to authorized users.

## Background

Luminal breast cancer, a molecular tumour classification subtype, is characterised by being estrogen receptor (ER) and/or progesterone receptor (PR) positive and responding well to endocrine therapy [1, 2]. Because overexpression of human epidermal growth factor receptor (HER2) worsens patient outcomes, among all subtypes, luminal HER2-negative carries the most favourable prognosis. The majority of patients with luminal HER2-negative tumours would be advised to receive only adjuvant endocrine therapy after surgery [2, 3]. However, there is a luminal HER2-negative subpopulation in which poorer outcomes can be expected with hormone therapy alone; these patients would benefit from chemotherapy. On the other hand, the significance of chemotherapy for patients with luminal tumours is now controversial in terms of both efficacy and adverse effects [2, 4, 5]. Thus, we often face difficulty in determining whether we should recommend (neo-) adjuvant chemotherapy to such patients in clinical practice.

Ki67, also known as MKI67, a nuclear protein associated with cellular proliferation, is a well-established marker for predicting the outcomes of patients with luminal breast cancer [6–10]. As a tumour with high Ki67 expression carries a poor prognosis, the expression level of this marker can have a major impact on treatment decisions particularly for patients with luminal HER2-negative tumours. According to biological differences, hormone receptor positive breast cancer is categorised into two groups: luminal A-like and B-like tumours with good and poor prognoses, respectively [1]. These two groups can be approximately distinguished based on low and high expressions of Ki67. Since the St. Gallen consensus in 2011, the well-known value of “14%” has been regarded as the Ki67 cut-off value for distinguishing between luminal A-like and B-like tumours and, as an index, it serves as the basis for making treatment decisions [2, 7, 8, 11]. Moreover, luminal tumours with high Ki67 expression reportedly respond well to chemotherapy [10, 12, 13], probably reflecting their high proliferative activity.

The aim of chemotherapy is to improve patient outcomes, as represented by overall survival. Thus, the only means of definitively evaluating treatments is to collect long-term postoperative outcome data but this would be very time-consuming, probably taking at least 10 years. To solve this problem, the effects of treatments are actually evaluated based on pathological findings in the NAC setting. Indeed, the chemo-effect, i.e. whether or not a patient has obtained pCR, has been validated as a surrogate marker of long-term survival [14, 15], although the significance of pCR might vary among luminal and other breast cancer subtypes [4, 5, 16].

Herein, we investigated Ki67 expression to predict the responses of luminal HER2-negative breast cancer patients to NAC, in order to identify a subpopulation potentially benefiting from chemotherapy.

## Methods

### Patients

We enrolled 114 patients with luminal HER2-negative invasive breast cancer, who underwent surgery after receiving NAC at our hospital during the 2006 through 2011 period. All tumours were ER positive and HER2 negative. We excluded HER2-positive cases from this study since their tumours have different biological characteristics, necessitating additional adjuvant treatments. As to NAC regimens, 108 of the 114 patients were given 4 cycles of CEF (C: cyclophosphamide 500 mg/m2, E: epirubicin 75 or 100 mg/m2, F: 5-FU 500 mg/m2), followed by taxanes (12 weeks of paclitaxel: 80 mg/m2 or 4 cycles of docetaxel: 75 mg/m2), prior to surgery. Five other patients received only CEF while one was given only a taxane, due to cardiac dysfunction. As to adjuvant treatment, 110 of the 114 patients (96%) had a history of adjuvant endocrine treatment after surgery according to menstruation status. We administered aromatase inhibitors to 75 patients, tamoxifen to 19 and tamoxifen with a luteinising hormone-releasing hormone analogue to 16. This study was carried out with approval from the ethics committee of Juntendo University Hospital (reference no. 21–570) and written informed consent was obtained from all participants.

### Pathological examination and immunohistochemistry

Pathological examinations were carried out by two pathologists at the Department of Human Pathology of Juntendo University. To avoid chemotherapy-related effects, we used biopsy specimens to examine biomarkers including Ki67. Biopsy specimen fixation was started within 1 minute after the biopsy, employing 15% buffered formalin, and was continued for 24 hr. Samples were then processed and embedded in paraffin. Sections 4 μm in thickness were cut and deparaffinised. Antigen retrieval was accomplished in citrate buffer solution (pH6.0) at 98°C for 45 minutes following endogenous peroxidase blocking with 0.3% hydrogen peroxide in methanol. Samples were incubated in monoclonal antibody against Ki67 (MIB-1, Dako, Denmark) (1:400) at 4°C overnight and then developed with 3,3′-diaminobenzidine. Cells positive for nuclear Ki67 were counted in at least 500 cancer cells in one hot spot for each of the biopsy specimens. ER and PR were judged to be positive when more than 10% of the nuclei of cancer cells were stained. HER2 was judged to be positive if more than 10% of tumour cells showed strong staining of the entire cell membrane, or HER2/neu gene amplification was confirmed by fluorescence in situ hybridisation. As indicated above, we excluded such HER2-positive tumours in this study. Nuclear grade was judged based on the modified Bloom-Richardson histologic grades [17]. Chemotherapy effects were determined employing operative specimens and we defined pCR as invasive nest disappearance based only on the primary breast tumour, i.e. without lymph node evaluation.

### Statistical analysis

Using JMP® 9 (SAS Institute Inc., Cary, NC, USA), we applied Fisher’s exact test to examine the independence of two categorical variables. For comparison of mean values such as patient age and tumour size, examinations of unpaired data were carried out with the two-sided Student’s *t*-test or Wilcoxon rank sum test, according to the data distribution. Kaplan-Meier curves were estimated and the logrank test was applied for comparisons of the survival distributions of two populations.

The optimal Ki67 cut-off value predicting pCR was determined using Receiver Operating Characteristic (ROC) curve analysis. The curve was created by plotting the true positive fraction (=sensitivity, equivalent to pCR rate herein) on the y-axis versus the false positive fraction (=1-specificity) on the x-axis for each Ki67 value tested in the 10% to 50% range. With this statistical method, the best possible prediction point is in the upper left corner, with coordinates of 0 and 1 in the ROC space, and is referred to as *the perfect classification*. We determined the optimal Ki67 value to be that nearest the perfect classification.

## Results

### Factors influencing chemo-effect

Clinicopathological features of patients and chemo-effects are presented in Table 1. All biomarkers were determined using biopsy specimens. The proportions of patients with each stage of breast cancer were; IIA: 42% (48 cases), IIB: 45% (51 cases), IIIA: 11% (12 cases) and IIIB: 3% (3 cases). The pCR rate was 10% (11 cases), which is approximately half of the average for all subtypes. The response of PR-negative tumours to NAC was significantly better than that of PR-positive tumours (p < 0.05). Ki67 was another factor related to the chemo-effect. Tumours with high Ki67 expression showed better responses to chemotherapy (p < 0.01) (Table 1 and Additional file 1: Figure S1), while no direct relationship was seen between PR status and Ki67 expression (Additional file 2: Figure S2).

**Table 1:** **Clinicopathological features of 114 patients and chemo-effects**

	pCR		non-pCR		p value
n	11		103		
Mean age (SD)	54.6	(16.4)	50.9	(11.1)	n.s.
Mean tumour size^a^ (mm) (SD)	35.9	(18.1)	36.0	(15.7)	n.s.
Histology					
Ductal	11		98		n.s.
Lobular	0		5		
High nuclear grade	27%	(3/11)	22%	(22/99)	n.s.
ER^b^	100%	(114/114)	100%	(114/114)	n.s.
PR^b^	45%	(5/11)	78%	(80/103)	<0.05
Mean Ki67 (%) (SD)^c^	43.0	(30.0)	33.6	(18.1)	<0.01

ER: estrogen receptor, PR: progesterone receptor, n.s.: not significant.

^a^clinical size measured by ultrasound before treatments.

^b^rates of positive (more than 10% of the nuclei of cancer cells) cases.

^c^dot chart shown in Additional file 1: Figure S1.

The NAC response was significantly better in patients with tumours highly expressing Ki67 (p < 0.01). PR-negative tumours also showed better responses to chemotherapy (p < 0.05).

### Ki67 cut-off value

**Figure 1:**
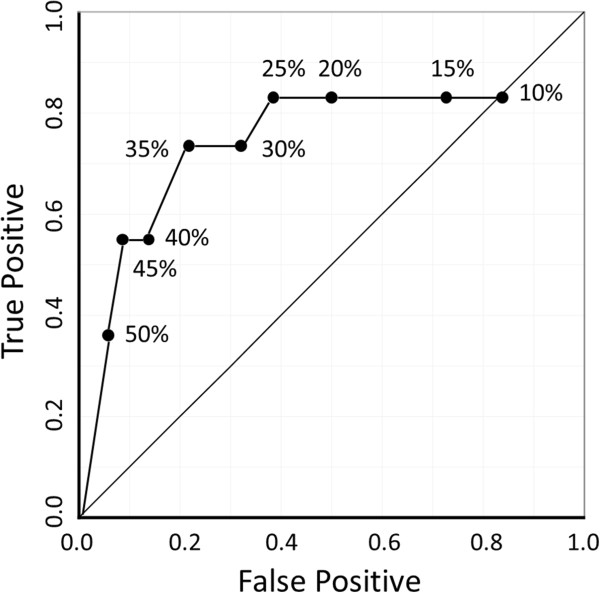
**ROC curve analysis revealed 35% to be the optimal Ki67 cut-off value for distinguishing between pCR and non-pCR cases.** ROC curve analysis revealed that, in the 10% to 50% Ki67 range, 35% was the optimal cut-off value for distinguishing pCR from non-pCR cases among our 114 patients. At 35%, the specificity and sensitivity were 0.79 and 0.73, respectively.

Next, using ROC curve analysis, we endeavoured to identify the Ki67 cut-off value which distinguishes pCR from non-pCR cases (Figure 1). The area under the curve (AUC), a diagnostic index, was 0.75, indicating this analysis to have moderate accuracy based on the AUC value being between 0.9 and 0.7 [18]. In the 10% to 50% range, testing in 5% increments, we identified the Ki67 value nearest the perfect classification, as this would be the most effective cut-off Ki67 value for predicting pCR. The plot with 35% as the Ki67 cut-off value, (coordinates 0.21 and 0.73) was closest to the perfect classification. Thus, we concluded 35% to be optimal Ki67 cut-off value for distinguishing pCR from non-pCR cases among our 114 patients (Specificity: 0.79, Sensitivity: 0.73). The pCR rates clearly differed between cases with Ki67 expressions above and below this cut-off value, at 27% and 4%, respectively.

### Patient outcomes

**Figure 2:**
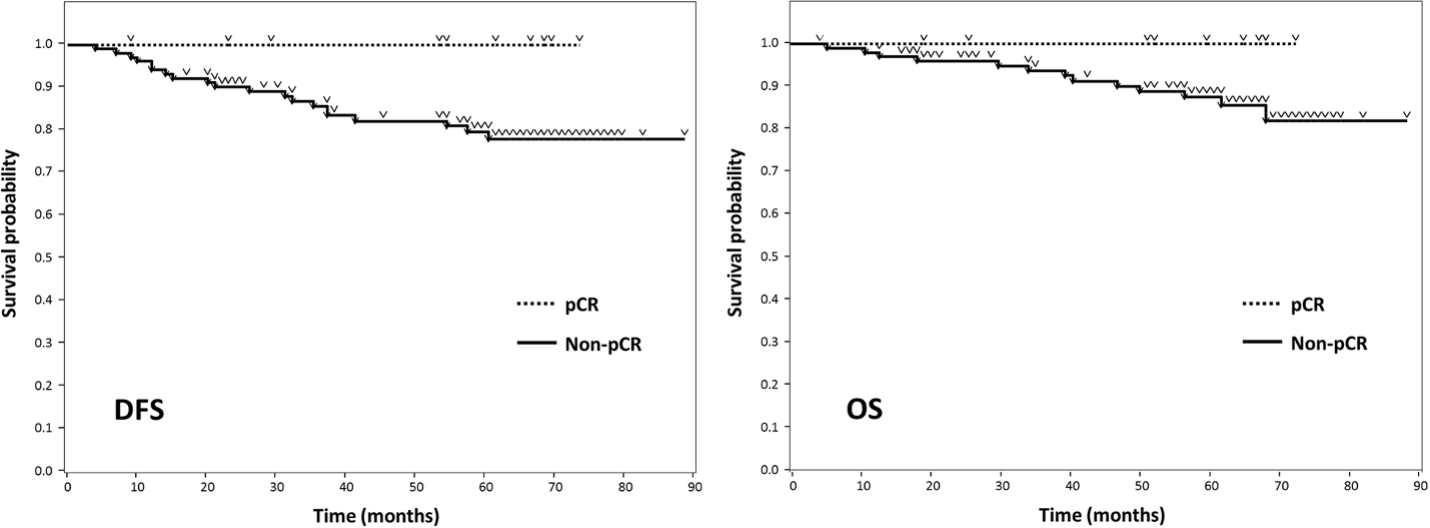
**Kaplan-Meier curves of 114 patient outcomes according to chemotherapy effect.** Twenty patients, none of whom had shown pCR, developed recurrent disease during the median 58-month follow-up period. Thirteen of these patients died due to breast cancer. The pCR group had higher rates of DFS and OS than the non-pCR group (100% vs 78.1%, 100% vs 87.7%, respectively at 60 months), although statistical significance could not be determined due to the lack of events in the pCR group.

**Figure 3:**
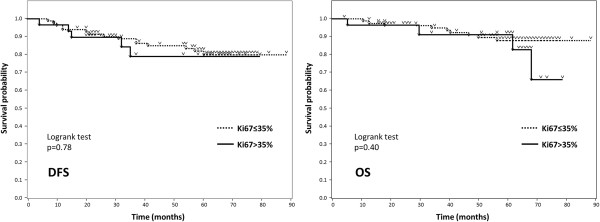
**Kaplan-Meier curves of 114 patient outcomes according to Ki67 expression (cut-off: 35%).** With Ki67 cut-off values ranging from 10 to 50%, there was no relationship between Ki67 expression and patient outcomes.

One hundred and ten patients (96%) were given adjuvant endocrine therapy after surgery. Among these 110, 75 patients had received aromatase inhibitors while the remainder were administered Selective Estrogen Receptor Modulators. During the median 58-month follow-up period (9–88 months), 20 patients (18%), none of whom had shown pCR, developed recurrent disease. Thirteen of these patients died of their breast cancer. When the outcomes were analysed according to chemo-effect, Kaplan-Meier curves showed that the pCR group had higher rates of disease-free survival (DFS) and overall survival (OS) than the non-pCR group (100% vs 78.1%, 100% vs 87.7%, respectively at 60 months), although statistical significance could not been determined because there were no events in the pCR group (Figure 2). Next, we assessed the relationship between Ki67 expression and patient outcomes. Kaplan-Meier curves with a 35% cut-off point for Ki67 are shown in Figure 3. Neither 35% nor any of the other Ki67 cut-off values (10-50%) showed a clinically meaningful relationship between Ki67 expression and either DFS or OS.

### Validation of the Ki67 cut-off value

**Figure 4:**
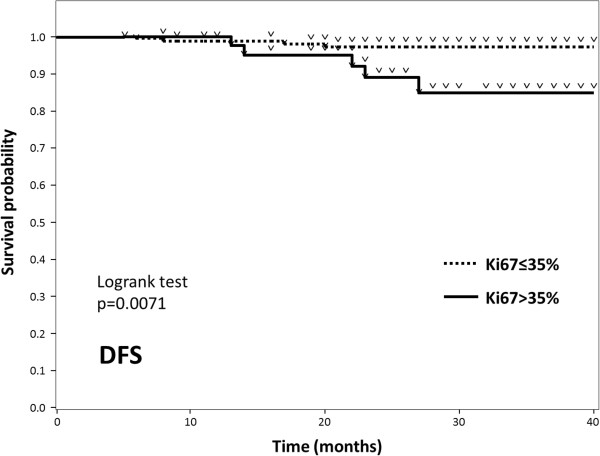
**Kaplan-Meier curves of DFS according to Ki67 (cut-off: 35%) in 196 HER2-negative luminal breast cancer patients.** Of these 196 patients, nine developed recurrent disease during the median 29-month follow-up period. Despite the short observation period, DFS differed significantly between patients whose tumours had Ki67 expression higher than 35% versus lower than 35% (p < 0.01).

Our results clearly showed a 35% cut-off value for Ki67 to distinguish pCR from non-pCR luminal breast cancer cases. To validate this cut-off value, we investigated patients with HER2-negative luminal tumours who underwent surgery at our institution during the 18-month period starting in April 2010, regardless of whether or not they received NAC. There were 196 patients and their clinicopathological features are shown in the Table 2. Compared to the 114 described above, this population includes relatively low-risk cases, i.e. 45% of these 196 patients had Stage I while the 114 all had stage II or III disease. Only 31% of this population (61 of 196) received (neo-) adjuvant chemotherapy plus endocrine therapy while 65% were given only endocrine therapy. Nine patients (5%) developed recurrent disease and two of them died during the median 29-month follow-up period (5–40 months). The observation period was, unfortunately, relatively short because only data from the time period when Ki67 examinations started to be routinely performed for all surgical cases in our hospital were available. Nonetheless, DFS differed significantly between patients who had tumours with Ki67 higher than 35% versus lower than 35% (p < 0.01) (Figure 4), while a similar but smaller difference was observed with a Ki67 cut-off value of 25% (p < 0.05) in those with values ranging from 10 to 50%.

**Table 2:** **Clinicopathological features and outcomes of 196 HER2-negative luminal breast cancer patients**

	Recurrence		No recurrence		p value
n	9		187		
Mean age (SD)	55.3	(13.9)	55.9	(12.1)	n.s.
Stage^a^					
I	2		87		
IIA or B	4		96		<0.01
IIIA or B	3		4		
Histology					
Ductal	7		161		
Lobular	2		11		n.s.
Others	0		15		
High nuclear grade	11%	(1/9)	4%	(7/181)	n.s.
High histological grade	22%	(2/9)	4%	(6/141)	n.s.
ER^b^	100%	(9/9)	100%	(187/187)	n.s.
PR^b^	67%	(6/9)	78%	(145/187)	n.s.
Mean Ki67 (%) (SD)	37.9	(21.7)	24.1	(18.5)	<0.05
(Neo-) adjuvant therapy					
Endocrine + chemotherapy	9		52		
Endocrine therapy only	0		127		
Chemotherapy only	0		1		
None	0		7		

n.s.: not significant.

^a^determined according to pathological findings.

^b^percentage of positive (more than 10% of the nuclei of cancer cells) cases.

There were 196 patients with HER2-negative luminal tumours who underwent surgery during the 18-month period starting in April 2010. Compared to the 114 described above, this population includes relatively low-risk patients, i.e. 45% of the 196 patients had Stage I while all of the 114 had stage II or III disease. (Only 31% of this population received NAC plus endocrine therapy).

## Discussion

In this study, the pCR rate was 10%, which is within the range established by previous studies (6-11%) focusing on HER2-negative and hormone receptor-positive patients [4, 19, 20]. Tumours with high Ki67 expression and/or PR-negativity showed a good response to chemotherapy. As illustrated in Additional file 2: Figure S2, there was no clear correlation between PR status and Ki67 expression. While PR-negative tumours might now have to be regarded as luminal B-like [21, 22], we did not differentiate these from PR-positive cases in the current study since this is a topic awaiting further careful consideration. There was no meaningful relationship between Ki67 expression and either DFS or OS. This is possibly because all patients enrolled in the present study received NAC, meaning that only relatively high-risk tumours were selected and also that some patients with tumours showing high Ki67 expression may have benefitted from salvage therapy with NAC and thus remained recurrence-free.

Our results support those of Fasching et al., who performed an elegant analysis of data from a large group of patients [20]. Employing two statistical models, they investigated the predictive and prognostic values of Ki67 in all subtype groups. Ki67 cut-off values between 36% and 40% were found to predict pCR to NAC for the hormone receptor-positive and HER2-negative breast cancer cases. The pCR group had more favourable outcomes, despite this population having a higher Ki67 proliferation rate. Another study from the GeparTrio trial similarly showed that tumours with Ki67 higher than 35%, as a group, had the best chemo-effect [23]. Our results are consistent with theirs, though we analysed Ki67 employing a different approach. In the current study, pCR was frequent in patients whose tumours showed Ki67 expression higher than 35% and all patients who obtained pCR remained recurrence-free. On the contrary, in the 196 patients recruited for validation, DFS was significantly lower for tumours with Ki67 higher than 35%. These results suggest that the population with Ki67 higher than 35% might benefit from chemotherapy, achieving improved survival, and this was the issue that we explored in the present study.

Obtaining pCR in luminal cases is sometimes questioned as von Minckwitz et al. indicated that pCR might be less significant than previously thought in luminal A-like and luminal B/HER2-positive-like cases [4]. However, our results do not necessarily conflict with the arguments of von Minckwitz and colleagues as our patient population included considerable numbers of luminal B-like cases, in which clinical benefit was clearly obtained in their study. The same logic can be applied to other reports showing the advantages of obtaining pCR in the luminal breast cancer population [16, 20]. We speculate that further clinically significant observations might be made in identifying a subpopulation among those with luminal HER2-negative tumours who could be salvaged by chemotherapy, rather than concentrating solely on distinguishing between luminal A-like and B-like tumours with Ki67 cut-off values such as 14%.

While our study has the limitations of being retrospective and having a relatively small number of subjects, it has the major advantage of consistency in immunostaining results. Using only biopsy specimens, that had been immediately fixed after sample collection, all processing steps and evaluations were performed in a single institution, an important feature of our study given that inconsistencies in the assessment of Ki67 among institutions have yet to be resolved [21, 24–26]. Evaluation employing biopsy specimens might be affected to some degree by heterogeneity within a single tumour, as compared to examinations based on whole operative specimens. However, given the goal of this study, we believe that avoiding the possible effects of NAC outweighs any possible disadvantages associated with tumour heterogeneity.

## Conclusion

Though it is necessary to examine long-term patient outcomes to obtain evidence supporting our results, we identified a Ki67 cut-off value, 35%, for selecting patients who would benefit from chemotherapy.

## Electronic supplementary material

Additional file 1: Figure S1: Dot chart of chemotherapy effects and Ki67 expression in 114 patients. (DOCX 46 KB)

Additional file 2: Figure S2: Dot chart of PR status and Ki67 expression in 114 patients. (DOCX 34 KB)
