# Supplementary figures and images for: Ki67 expression and the effect of neo-adjuvant chemotherapy on luminal HER2-negative breast cancer
Source: BMC Cancer. 2014 Jul 30;14:550. doi: 10.1186/1471-2407-14-550 (PMC4124146; doi:10.1186/1471-2407-14-550)

Additional file 1: Figure S1. Dot chart of chemotherapy effects and Ki67 expression in 114 patients


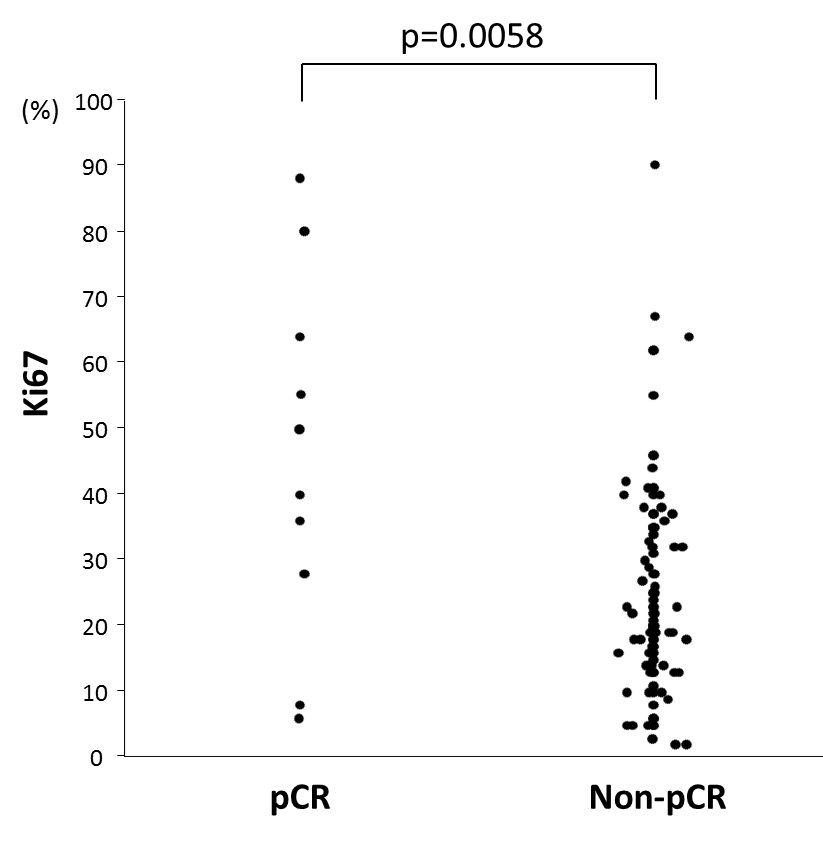

Supplement: Supplementary file 1 — Additional file 1: Figure S1: Dot chart of chemotherapy effects and Ki67 expression in 114 patients. (DOCX 46 KB) [file 12885_2013_4730_MOESM1_ESM.docx]

Additional file 1: Figure S2. Dot chart of PR status and Ki67 expression in 114 patients


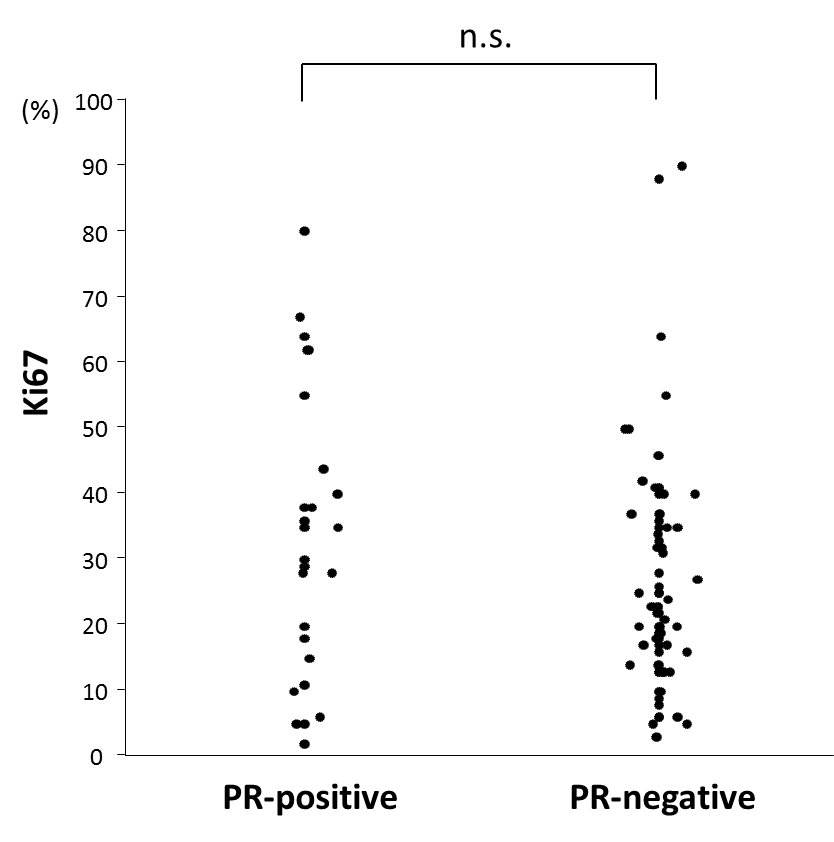

Supplement: Supplementary file 2 — Additional file 2: Figure S2: Dot chart of PR status and Ki67 expression in 114 patients. (DOCX 34 KB) [file 12885_2013_4730_MOESM2_ESM.docx]
